# Early intravenous tranexamic acid intervention reduces post-traumatic hidden blood loss in elderly patients with intertrochanteric fracture: a randomized controlled trial

**DOI:** 10.1186/s13018-020-02166-8

**Published:** 2021-02-03

**Authors:** Huixu Ma, Hairuo Wang, Xiaotao Long, Zexiang Xu, Xiaohua Chen, Mingjin Li, Tao He, Wei Wang, Lei Liu, Xi Liu

**Affiliations:** 1 Department of Orthopaedics, Chongqing General Hospital, Chongqing, 400021 People’s Republic of China; 2 Department of Orthopedics, National Clinical Research Center for Geriatrics, West China Hospital, Sichuan University, Chengdu, 610041 People’s Republic of China; 3 Department of Radiology, Chongqing Traditional Chinese Medicine Hospital, Chongqing, 400022 People’s Republic of China

**Keywords:** Tranexamic acid, Hip fracture, Elderly, Blood loss

## Abstract

**Purpose:**

Elderly patients with intertrochanteric fractures exhibit post-traumatic hidden blood loss (HBL). This study aimed to evaluate the efficacy and safety of reducing post-traumatic HBL via early intravenous (IV) tranexamic acid (TXA) intervention in elderly patients with intertrochanteric fracture.

**Methods:**

A prospective randomized controlled study was conducted with 125 patients (age ≥ 65 years, injury time ≤ 6 h) who presented with intertrochanteric fracture from September 2018 and September 2019. Patients in the TXA group (*n* = 63) received 1 g of IV TXA at admission, whereas those in the normal saline (NS) group (*n* = 62) received an equal volume of saline. Hemoglobin (Hgb) and hematocrit (Hct) were recorded at post-traumatic admission (PTA) and on post-traumatic days (PTDs) 1–3. HBL was calculated using the Gross formula. Lower extremity venous ultrasound was performed to detect venous thrombosis.

**Results:**

Hgb on PTDs 2 and 3 was statistically higher in the TXA group than in the NS group. Hct and HBL on PTDs 1–3 were significantly less in the TXA group compared to the NS group. Preoperative transfusion rate was significantly lower in the TXA group compared with the NS group. There was no difference between the two groups with regard to the rates of complications.

**Conclusion:**

Early IV TXA intervention could reduce post-traumatic HBL and pre-operative transfusion rate in elderly patients with intertrochanteric fractures without increasing the risk of venous thrombosis.

## Introduction

Globally, hip fracture is a frequent cause of morbidity and mortality, particularly among elderly people [1]. Elderly patients with osteoporosis exhibit a high incidence of intertrochanteric fractures, such that one in three patients die from various complications caused by fracture within 1 year after injury [2].

Recent studies have confirmed that elderly patients with intertrochanteric fractures exhibit preoperative non-negligible hidden blood loss (HBL), which can impact functional outcomes and increase mortality by lowering hemoglobin (Hgb) levels. Patients with intertrochanteric fractures show greater blood loss than those with femoral neck fractures and have a higher transfusion rate [3]. Thus, a method to effectively reduce the amount of HBL in elderly patients with intertrochanteric injuries and reduce the incidence of related complications caused by HBL is worth further study.

TXA is a synthetic analog of an amino acid with biological activity that inhibits plasminogen from dissolving clots; such activity can reduce blood loss and transfusion requirements [4]. Previous studies have shown that TXA reduces total blood loss and the requirement for transfusion in hip arthroplasty and hip fracture surgery [5, 6]. However, the majority of these studies were focused on the hemostatic effect of TXA on postoperative visible blood loss and HBL in hip fracture surgery, rather than on preoperative HBL. Recently, studies describing HBL during the perioperative period following intertrochanteric fractures have been increasing in number [7, 8]. Few studies have investigated whether TXA can reduce post-traumatic HBL in elderly patients with intertrochanteric fractures [7].

All the above, we conducted the present study in patients with intertrochanteric fractures over 65 years old, and all the patients were randomized into two groups (TXA group: IV TXA; NS group: IV NS).

The hemostatic efficacy of the above two groups was compared and analyzed, based on post-traumatic HBL, the pre-operative transfusion (POT) rate. In addition, the incidence of DVT and PE was recorded and compared to evaluate the safety of TXA intervention in patients over 65 years old. As the early IV TXA intervention, we hypothesized that early TXA intervention would achieve less post-traumatic HBL and reduce the POT rate, without increasing the risk of DVT.

## Materials and methods

### Study design and patients

This prospective randomized controlled trial was conducted at the Department of Orthopaedics of our hospital and registered in the Chinese Clinical Trial Registry (ChiCTR-1800017761). The study was conducted in accordance with the principles of the Declaration of Helsinki and was approved by the local Ethics Committee of our hospital (No. S2017-022), and written informed consent and research authorizations were obtained from all participants. All elderly patients with intertrochanteric fractures (age ≥ 65 years, injury time ≤ 6 h) who underwent treatment at the Department of Orthopaedics in the Chongqing General Hospital between September 2018 and September 2019 were prospectively investigated. Inclusion criteria were (1) patients diagnosed with fresh unilateral intertrochanteric fracture and fracture classified according to AO type according to computed radiography or computed tomography and (2) patients aged ≥ 65 years with intertrochanteric fracture and injury time ≤ 6 h. Exclusion criteria were (1) patients with injury time > 6 h; (2) those with open fractures, other parts of the body with hemorrhagic wounds, or other areas with bleeding disorders (such as gastrointestinal bleeding); (3) those who exhibited additional fresh fractures in other body parts; (4) patients with recent or ongoing thromboembolic events (deep venous thrombosis, pulmonary embolism, arterial thrombosis, or cerebral thrombosis stroke); (5) patients who were recently taking or who were taking anticoagulation therapy including vitamin K-antagonists, direct thrombin inhibitors, direct factor X-a inhibitors, and platelet aggregation inhibitors; (6) patients with disseminated intravascular coagulation or patients had hepatic or renal diseases with impairment of coagulation function; (7) those receiving conservative treatment; and (8) those with TXA allergy or allergies.

### Drug delivery and randomization

Drug delivery and randomization recruited patients were randomly allocated into two groups (TXA group: IV TXA; NS group: IV NS) based on a computer-generated randomization list, which was generated with the use of Randomization.com. The randomization was prepared by a statistician who was not involved in this clinical trial. Patients in the TXA group received i.v. TXA (0.5 g; Ruiyang Pharmaceutical Co., Ltd., Shandong, China) 1 g (200 mL) immediately post-traumatic admission (PTA), and those in the NS group received 200 mL of NS (i.v) immediately PTA. All patients received low molecular weight heparin sodium anticoagulation 6 h after injury.

### Outcome measurements

Patient demographic and clinical characteristics, including sex, age, weight, and height, were immediately recorded after admission. Routine blood examination was performed at PTA and on post-traumatic days (PTDs) 1–3 to determine hemoglobin (Hgb) and hematocrit (Hct) levels.

The primary outcome measures include post-traumatic HBL, the pre-operative transfusion (POT) rate, Hgb drop, Hct change, and the incidence of DVT (lower limb venography was performed at the time of 3 months of follow-up if a patient exhibited symptoms of venous thromboembolism) and PE (computed tomography was taken to examine PE if any suspicious symptom were complained). We used the Gross equation [9] to calculate post-traumatic HBL: HBL (ml) = PBV × (Hct_1_ − Hct_2_), Hct_1_: Hct level at admission, and Hct_2_: Hct level at a given post-traumatic time point. Patient blood volume (PBV) was calculated using the formula of Nadler et al. [10] as follows: PBV in males = 0.3669 × height (m)^3^ + 0.03219 × weight (kg) + 0.6041, PBV in females = 0.356 × height (m)^3^ + 0.3308 × weight (kg) + 0.1833. The criterion of blood pre-operative transfusion (POT) was as an Hgb level of < 80 g/L or symptomatic anemia (light-headedness, palpitation, or shortness of breath not associated with other etiologies) in a patient with an Hgb level of 80–100 g/L [11].

The secondary outcomes included the length of admission to operation, length of hospital stay, and complications (cardiac infarction, ischemic cerebral infarction, stroke, respiratory infection, and renal failure).

### Sample-size calculations

We assumed that this IV-TXA application should reduce hidden blood loss more than 20% compared with control group. Setting the pre-study power of test (*β*) as 0.9, significant difference (*α*) as 0.05, and standard effect size of 0.65 indicated that 51 patients were required for each group. To compensate for the expected dropouts (20%), 61 patients per group were planned to include in this study. Calculations were performed with G*Power 3.1.

### Statistical analysis

Data were analyzed using SPSS 21.0 statistical software (SPSS Inc., Chicago, IL, USA), and all relevant data were assessed for normality. Continuous data were analyzed using independent sample *t* tests, and the nonparametric alternative (Mann-Whitney *U* tests) was used where data were not normally distributed. A chi-square test or Fisher’s exact test for difference in proportions was used to estimate differences between groups in categorical variables. The difference was considered statistically significant if *P* < 0.05.

## Results

**Fig. 1:**
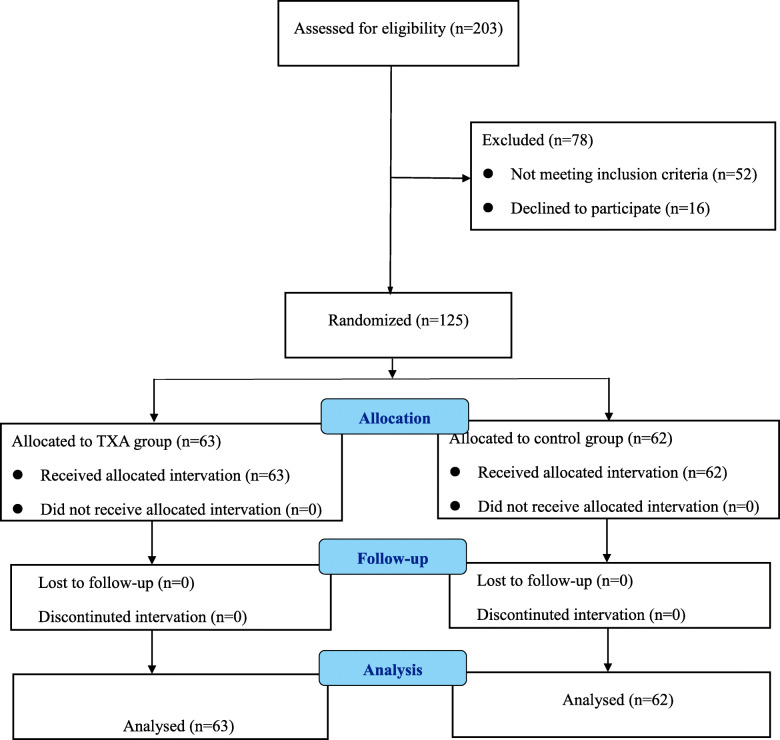
Flowchart of the inclusion and exclusion of the patients

Totally, 203 patients with intertrochanteric fractures (age ≥ 65 years) were screened for participation in our trial. However, 78 patients were excluded from the study, and the remaining 125 patients (43 males and 82 females) underwent randomization into two groups. The supplemental TXA group included 63 patients, and the NS group included 62 patients (Fig. 1). There was no demographic difference between the two groups (Table 1).

**Table 1:** Baseline characteristics

Variable	TXA group (n = 63)	NS group (*n* = 62)	*P* value
Female (*n*, %)	42 (66.67)	40 (64.52)	0.81
Age (year)	78.05 ± 7.62	78.66 ± 6.95	0.64
BMI (kg/m^2^)	22.27 ± 3.03	22.19 ± 2.87	0.89
AO fracture classification (A1/A2/A3)	21/26/16	20/28/14	0.74
ASA classification, I–II/III–IV	22/41	20/42	0.89
Length of trauma to admission (h)	2 (1 to 6)	2 (1 to 6)	0.45
Length of admission to randomization (h)	1.5 (1 to 2.5)	2 (1 to 2.5)	0.09
Closed reduction with PFNA (*n*, %)	57 (90.5)	58 (93.5)	0.65

Values are *n* (%) or mean ± SD. Categorical data were compared among groups using the chi-squared test

*BMI* body mass index, *PFNA* proximal femoral nail antirotation

Primary outcome data and complications of all the groups were presented in Tables 2 and 3. As Table 2 showed, Hgb level was lower in the TXA group compared to the NS group on PTD 1; however, these were not significantly different. Hgb levels were significantly higher in the TXA group compared with the NS group on PTD 2 and PTD 3, respectively. Hct level was significantly reduced in the TXA group compared to the NS group on PTD 1, PTD 2, and PTD 3. The mean HBL were significantly reduced in the TXA group compared to the NS group on PTD 1, PTD 2, and PTD 3, respectively. The preoperative transfusion (POT) rate in the TXA group (2.0 U packed RBC in 3 patients on PTD 4, 4.0 U packed RBC in 4 patients on PTD 5) was significantly lower compared to the NS group (2.0 U packed RBC in 10 patients on PTD 3, 4.0 U packed RBC in 4 patients on PTD 4).

**Table 2:** Comparison of post-traumatic outcomes between the TXA and NS groups

Variable	TXA group (*n* = 63)	NS group (*n* = 62)	*P* value
Hgb (g/L)			
Hgb PTA	115.60 ± 4.64	116.05 ± 7.16	0.68
Hgb PTD 1	108.30 ± 4.92	106.44 ± 6.11	0.07
Hgb PTD 2	104.44 ± 6.49	100.05 ± 5.93	<0.001
Hgb PTD 3	100.10 ± 6.97	88.29 ± 6.88	<0.001
Hct (%)			
Hct PTA	43.52 ± 2.22	42.92 ± 2.02	0.11
Hct PTD 1	38.60 ± 2.63	37.66 ± 2.41	0.04
Hct PTD 2	35.40 ± 3.21	31.35 ± 2.47	<0.001
Hct PTD 3	33.35 ± 3.47	26.65 ± 2.73	<0.001
HBL (mL)			
HBL PTD 1	172.87 ± 24.53	183.55 ± 20.58	0.01
HBL PTD 2	217.79 ± 32.51	288.16 ± 32.32	. < 0.001
HBL PTD 3	253.97 ± 35.98	407.50 ± 32.48	<0.001
POT rate (*n*, %)	7 (11.11)	14 (22.58)	.036
POT units (U)	22	36	-
Length of admission to operation (h)	70 (10 to 144)	72 (12 to 160)	0.84
Length of hospital stay (d)	7 (4 to 10)	7 (4 to 11)	0.61

Values are *n* (%) or mean ± SD. Categorical data were compared among groups using the chi-squared test

*Hct* hematocrit, *Hgb* hemoglobin, *HBL* hidden blood loss, *PTA* post-traumatic admission, *PTD* post-traumatic day, *POT* pre-operative transfusion

**Table 3:** Complications

Complicatios	TXA group (*n* = 63)	NS group (*n* = 62)	*P* value
Venous thrombosis (*n*)	9 (14.3%)	8 (12.9%)	0.515
DVT (*n*)	0	0	–
PE (*n*)	0	0	–
Respiratory infection (*n*)	10 (15.9%)	12 (19.4%)	0.517
ICI (*n*)	0	0	–
Stroke (*n*)	0	0	–
Cardiac infarction (*n*)	0	0	–
Acute renal failure (*n*)	0	0	–

Values are *n* (%) or mean ± SD. Categorical data were compared among groups using the chi-squared test

*DVT* deep venous thrombosis, *PE* pulmonary embolism, *ICI* ischemic cerebral infarction

There were no systemic complications related to TXA intervention and no cases of DVT in the lower extremities. As Table 3 showed, 9 patients developed venous thrombosis in the TXA group; 5 of these patients exhibited calf muscle thrombosis and 4 exhibited posterior tibial vein thrombosis. In the NS group, 8 patients developed venous thrombosis; all of these patients exhibited calf muscle venous thrombosis. No significant difference was found between the two groups in the incidence of lower extremity venous thrombosis.

As for the secondary outcomes presented in Tables 2 and 3, ten patients developed in the TXA group and twelve patients in the NS group developed respiratory infection. The differences were not statistically significant. No PE, cardiac infarction, ischemic cerebral infarction, stroke, and acute renal failure were observed in either group during the 3-month follow-up period (Table 3). Other complications were not reported. There were no significant differences of the median length of admission to operation and hospital stay between the two groups (Table 2).

## Discussion

The most important finding of this study was that the early application of intravenous TXA supplements can reduce the post-traumatic HBL, POT rate, and without increasing the incidence of DVT.

A previous study has shown that the mortality of hip fracture in elderly patients was related to the timing of surgery, sex, or preoperative/postoperative anemia [12]. In addition, perioperative Hgb and Hct levels have implications for outcomes because patients with hip fractures are typically frail and elderly and are, thus, particularly prone to anemia and hypovolemia [13]. Another study has shown obvious HBL in the perioperative period in patients with hip fractures [3]; moreover, the amount of HBL after hip fracture in elderly patients gradually increases with time [8]. Anemia due to HBL could prolong wound healing and hospitalization times, increase the incidence of postoperative pulmonary and cerebral edema, and cause several other problems [6].

Two consecutive global multi-center studies showed that the early application of TXA effectively reduces the rates of mortality due to traumatic bleeding [14, 15]. Previous studies have shown that TXA reduces postoperative HBL associated with intertrochanteric fractures [16], total knee arthroplasty [17], and extracapsular fracture of the hip [6]; these effects occurred without increasing the risk of postoperative venous thromboembolism. However, it was unclear whether TXA could reduce post-traumatic HBL in patients after intertrochanteric fractures. Based on these previous findings, our present study evaluated early TXA intervention (i.e., immediately after admission) in elderly patients with intertrochanteric fractures and showed that, compared with NS, early TXA intervention (≤ 6 h) controlled the reduction of post-traumatic Hgb and Hct levels in elderly patients with intertrochanteric fractures. The amount of HBL was significantly lower in the TXA group than in the NS group on PTDs 1–3. Thus, early TXA intervention in elderly patients with intertrochanteric fractures could effectively reduce the amount of post-traumatic HBL compared with that observed in the NS group. Furthermore, our data suggest that early TXA intervention could reduce the number of orthopedic patients who require transfusion: early intervention of 1 g of TXA reduced the transfusion rate from 22.58 to 11.11%. This might substantially reduce healthcare costs and for such elderly patients with intertrochanteric fractures.

Several studies have demonstrated that delaying surgery for more than 24 h increases mortality [18, 19]. Other studies have demonstrated no significant difference in the mortality of patients in whom surgery was delayed by up to 3 days [20]. Previous study reported that the frequent reasons for delaying surgery from acute medical comorbidity included active chest infection, anemia, electrolyte imbalance, heart failure, and others [21]. Although our results showed that the early intervention TXA can effectively reduce the post-traumatic HBL and the pre-operative transfusion rate, but the significant differences of the median length of admission to operation and hospital stay between the two groups were not found. Because there are other factors that affect the delaying surgery and the length of hospital stay, such as the patients who had initially declined surgery but later changed their minds [22, 23].

Although numerous studies have reported the safety of using TXA with routine methods [17, 24], the safety of supplemental dosages of TXA is still a matter of debate, as the study has reported that there was an increased risk of DVT for higher doses of TXA or prolonged use [25]. However, currently, there is no consensus on whether the use of TXA increases the risk of postoperative lower limb DVT [4]. In this study, however, no significant difference was found between the two groups in the incidence of venous thrombosis in the lower extremities. The incidence of venous thrombosis in our study was comparable with the previous study [26].

Although this study was carefully designed, there were still several limitations. First, the sample size was relatively small, and a large-scale study is required to clearly assess the safety of medication in this population, but the sample size calculations showed that our small sample was sufficient. Second, this study solely focused on a short follow-up period, which may have been insufficient to assess the clinical efficacy and safety of this treatment. Finally, this study only used a single instance of early TXA intervention; further sequential interventions will require an in-depth study and will be reported later.

## Conclusion

In conclusion, our findings suggest that early IV TXA intervention could reduce post-traumatic HBL and pre-operative transfusion rate in elderly patients with intertrochanteric fractures without increasing the risk of venous thrombosis.
